# Azoospermia and embryo morphokinetics: testicular sperm-derived embryos exhibit delays in early cell cycle events and increased arrest prior to compaction

**DOI:** 10.1007/s10815-018-1183-8

**Published:** 2018-05-21

**Authors:** Nina Desai, Pavinder Gill, Nicholas N. Tadros, Jeffrey M. Goldberg, Edmund Sabanegh, Tommaso Falcone

**Affiliations:** 1 Cleveland Clinic, Department of Obstetrics and Gynecology, Division of Reproductive Endocrinology and Infertility, 26900 Cedar Road, Beachwood, OH 44122 USA; 2 Division of Urology, Southern Illinois University, PO Box 19665, Springfield, IL 62794 USA; 3 Cleveland Clinic, Department of Urology, Glickman Urological and Kidney Institute, 26900 Cedar Road, Beachwood, OH 44122 USA

**Keywords:** Time-lapse, morphokinetics, Azoospermia, Testicular sperm, Epidiymal sperm, Blastocyst, Genome activation, compaction

## Abstract

**Purpose:**

Sperm play an essential role in embryonic genome activation and embryonic progression to blastocyst. In the present work, we focus on development of embryos created as a result of ICSI with testicular or epididymal sperm from azoospermic males and compare this to outcomes from normospermic males. The objective of this study was to determine if sperm origin influences clinical outcomes, the kinetics of embryo development, or the incidence of cleavage anomalies and multinucleation.

**Methods:**

A total of 93 consecutive intracytoplasmic sperm injection cycles (ICSI) performed for 83 couples were included in this study. Observations were made on 594 fertilized oocytes cultured in the EmbryoScope using time-lapse microscopy (TLM). Epididymal sperm (*n* = 29) cycles or surgically retrieved sperm from the testis (TESE; *n* = 37 cycles) of men with either obstructive (OA) or non-obstructive azoospermia (NOA) were used to inject oocytes. A further 27 ICSI cycles were performed using ejaculated sperm from normospermic males, designated as our control sperm (CS) group. Kinetic data and cycle outcomes were retrospectively analyzed.

**Results:**

The clinical pregnancy rate was not different between the three groups (TESE 51.4%, PESA 57.7%, and CS 59.3%). A non-significant decrease was observed in both implantation (30.9%) and live birth rate (43%) with TESE as compared to PESA (35.3%, 58%, respectively) and CS groups (45.1%, 56%, respectively). Failure to compact was significantly higher amongst TESE-NOA embryos (35.2%; *P* < 0.001) as compared to TESE-OA (4%), PESA (9%), and CS (3.8%) embryos. The two points at which TESE-derived embryos (both NOA and OA) behaved most differently from PESA and CS embryos was at cc2 (t3-t2; time to initiation of the second cell cycle) and tSB (time to start of blastulation). A significantly lower percentage of TESE embryos exhibited kinetics typically ascribed to high quality embryos with the greatest developmental potential. Finally, the incidence of direct uneven cleavage (DUC) was observed to be significantly higher after ICSI with sperm retrieved from azoospermic males.

**Conclusions:**

TLM allowed a more in depth comparison of paternal influence on embryo morphokinetics and helped to identify specific differences in cell cycle kinetics. TESE-NOA embryos exhibited a higher incidence of compaction failure.

## Introduction

Approximately one third of couples seeking infertility treatment have male factor as their primary indication. Azoospermia, defined as the complete absence of sperm in the ejaculate, accounts for ~ 10% of these cases. Intracytoplasmic sperm injection (ICSI) with sperm surgically retrieved from either the epididymis or testis has dramatically altered the prognosis for successful pregnancy in couples with severe male factor infertility, including azoospermia.

The underlying causes of male factor infertility are varied and likely influence the severity of impairment. Specific deletions on the long arm of the Y chromosome, known as the AZF (azoospermia factor) region are clinically important due to their association with failure or disruption of spermatogenesis [1–3]. Deletions on the Y chromosome have been associated with 15–20% of cases of azoospermia or severe oligospermia. Amongst azoospermic men, 10–15% will have an abnormal number of chromosomes. Chromosomal structural anomalies like translocations are ten times more likely in infertile men compared to the general population [4]. Autosomal inversion of chromosome 9 is especially relevant to male infertility and presents in 3–5% of such patients. Congenital absence of the vas deferens is most often associated with mutations within the cystic fibrosis gene. Other causes of azoospermia include endocrine disruptions, environmental factors, varicoeles, and chemical exposure.

Understanding reproductive failures as well as fertility outlook after therapeutic intervention are vital areas of research. Mitotic potential and nuclear syngamy of the embryo are paternally inherited [5]. Proper sperm aster formations followed by fusion of male and female pronuclei are all critical to the fertilization process. The sperm centrosome must replicate, divide, and organize formation of the mitotic spindle, necessary for subsequent cleavage [5–8]. Sperm also provide oocyte-activating factor, responsible for calcium oscillations leading to initiation of the mitotic cycle [9, 10]. Paternal contribution is also pivotal for activation of the embryonic genome and enabling blastocyst formation to proceed [11–13].

A high frequency of chromosomal abnormalities has been observed in testicular sperm from men with severe male factor infertility [14]. There is some evidence suggesting that azoospermia and consequent use of surgically retrieved testicular sperm results in lower fertilization rates, impaired embryonic development or lower embryonic implantation [15–19]. But a recent meta-analysis of 10 published studies (734 cycles) suggests that in men with similar etiology, sperm origin (whether testis or epididymis) does not seem to affect clinical outcomes [20].

Integration of time-lapse microscopy (TLM) in to in vitro fertilization laboratories allows more in depth analysis of embryonic development than was previously possible with conventional once daily observational checkpoints at 18, 42, 66, 90, and 114 h post-insemination. Quantitative assessment of sperm-associated influence on cell cycle parameters as well as detection of transient morphological attributes may help us better gauge paternal effects on embryo development. Data from TLM studies have demonstrated that cell cycle timings for early cleavage events as well as compaction and blastulation are correlated to embryo implantation potential [21–26]. Optimal timings for specific kinetic endpoints and selection algorithms have been proposed to help identify embryos with the highest implantation potential that on transfer are most likely to result in pregnancy. Sperm origin and paternal effects have heretofore not been central to these studies.

In the present work, we focus on development of embryos created as a result of ICSI with testicular or epididymal sperm from azoospermic males and compare this to outcomes from normospermic males. The objective of this study was to determine if sperm origin influences clinical outcomes, the kinetics of embryo development, or the incidence of cleavage anomalies and multinucleation.

## Materials and methods

### Study design

The study population consisted of women undergoing intracytoplasmic sperm injection (ICSI) with epididymal or testicular-derived sperm at the Cleveland Clinic between January 2013 and September 2015. For comparison purposes, we also included a control group of women undergoing ICSI during this same interval using ejaculated sperm with normal semen parameters, based on the World Health Organization 2016, 5th edition guidelines (> 15 M/ml, > 40% motility, > 4% normal forms). We further limited this patient subset to only tubal factor patients, to minimize female factor etiology that could possibly influence oocyte and/or embryo quality. A total of 93 consecutive cycles with 594 cultured zygotes were analyzed. Morphokinetic data from zygotes cultured in the EmbryoScope time-lapse chamber were retrospectively analyzed. Data collection for this study was approved by our Institutional Review Board (IVF data registry IRB # 5251 and Embryoscope data registry IRB# 14-566). This study was performed within the guidelines established by the Cleveland Clinic Institutional Review Board.

### Ovarian stimulation

Ovarian stimulation protocol selection was based on patient age, serum anti-Mullerian hormone levels, antral follicle counts, and prior response to gonadotropins. Women were treated with either a GnRH (gonadotropin releasing hormone) agonist or antagonist to suppress ovulation until follicle maturity was attained. Recombinant FSH, with or without urinary menotropins, was used for ovarian stimulation. Final follicular maturation was triggered with human chorionic gonadotrophin (hCG) and/or a GnRH agonist when at least two lead follicles measured 18 mm in mean diameter. Oocytes were collected 36 h later by transvaginal ultrasound-guided needle aspiration of follicles.

### Oocyte insemination, embryo culture, and transfer

Cumulus: oocyte complexes were cultured in HTF medium (Life Global; Guilford, CT) supplemented with 10% human serum albumin (Cooper-Surgical, Trumball, CT) under an oil overlay at 37 °C with 6% CO_2_ and air for 2–3 h. Cumulus cells surrounding the oocytes were then removed using enzymatic digestion with hyaluronidase (Cooper-Surgical). Conventional density gradient centrifugation was used to isolate sperm from ejaculated semen samples in our control sperm (CS) group.

Intracytoplasmic sperm injection (ICSI) was performed on metaphase II (MII) oocytes using either epididymal, testicular, or ejaculated sperm. Oocytes were checked for fertilization 16–18 h post-insemination. Normally fertilized zygotes were moved to individual wells of one or more EmbryoSlides (VitroLife). The EmbryoSlide containing 12 wells was prepared by filling each well with 25 μl of growth medium and overlaying with 1.4 ml of washed oil (LifeGlobal). EmbryoSlides containing zygotes were placed in the EmbryoScope and cultured for up to 6 days at 37 °C with 6% CO_2_ and 6% O_2_.

Transfer day was determined primarily by number of zygotes. Patients with lower zygote numbers (≤ 5) were generally scheduled for day 3 transfers. All other patients had their embryos cultured to day 5 before transfer. Number of embryos transferred was based on patient preference, quality, and previous infertility history. Transfers were performed under ultrasound guidance using a Wallace catheter. Serum hCG levels were measured 15 days after the embryo transfer. Clinical pregnancy was confirmed by the presence of a fetal heart on ultrasound examination at six to 8 weeks of pregnancy.

### Epididymal and testicular sperm retrieval

Epididymal sperm were obtained by percutaneous epididymal sperm aspiration (PESA). This procedure was performed under conscious sedation. A 23-gauge needle was passed through the scrotal skin and directly into the epididymis to aspirate epididymal fluid with negative pressure. Sperm aspirates were examined by the fertility laboratory for the presence of motile/twitching sperm.

Testicular sperm were surgically retrieved using a microscope-assisted testicular sperm extraction procedure (microTESE) in the operating room. After opening the scrotum, the tunica vaginalis was identified and incised to expose the testis. The operating microscope was then brought over the field and a small incision was made into the testis away from any obvious blood vessels. Gentle traction was used to expose a wide swath of seminiferous tubules which were then sampled. This procedure was repeated in different parts of the testis and/or the contralateral testis if sperm was not initially found. Six patients had conventional testicular sperm extraction without microscopic assistance, where smaller amounts of tissue were extracted. Excised tissue was placed in HTF medium and transported to the fertility laboratory for processing, assessment, and cryopreservation. Testicular tissue was ground in Eppendorf tubes using a Kontes pestle to release sperm. Five-microliter drops of the testicular specimen were assessed microscopically for presence of sperm. The number of motile/twitching sperm per high powered field (hpf) prior to cryopreservation was recorded.

All epididymal and testicular sperm specimens were retrieved before the planned ICSI cycle and cryopreserved. For cryopreservation, PESA and TESE samples were diluted 1:1 with test yolk buffer-glycerol cryoprotectant (Irvine Scientific, Irvine CA) and aliquotted in to cryovials. Vials were vapor frozen for 30 min prior to immersion in liquid nitrogen. On the morning of the egg retrieval, vials were thawed and examined for the presence of motile sperm. Specimens with at least one motile sperm per hpf were washed by centrifugation (200*g*, 10 min) and sperm pellets were re-suspended in culture media. With more severely compromised samples centrifugation was avoided. Motile or twitching sperm were identified and moved to cryoprotectant-free media drops using an ICSI needle. In such cases, an additional embryologist was assigned to help search through multiple vials for sperm. Immotile sperm were not used for ICSI.

### Time-lapse analysis and embryo grading

The EmbryoScope image acquisition system was set to capture high contrast 200× images from 5 to 7 focal planes for each embryo, every 15 min during the entire culture interval. All embryos were assessed daily by viewing time-lapse video footage and monitoring specific cell cycle events as well as embryo morphology. Cleavage stage embryos were assessed for cell stage, percent fragmentation, and blastomere symmetry. Embryos were screened for multinucleation and anomalous division patterns such as reverse cleavage and direct uneven cleavage (cleavage from 1 to 3 cell or 2 to 5 cell). From day 3 onwards, embryos were monitored for increase in cell to cell adherence between blastomeres leading to cell merging and compaction. Embryos were scored as either compacting (CP) if 2–3 cells were merged or else morula, when over 90% of cells had merged, forming a tight ball of cells. Blastocysts were evaluated for expansion, inner cell mass development, and trophectoderm appearance at 114 h (day 5) and 138 h (day 6).

Timing of specific cleavage events were calculated and expressed as hours (h) post-insemination, with start of ICSI being used as t0. Time to two-cell t2, t3, t4, t5, t8, t9+, compaction (CP), morula (tM), start of blastulation (tSB), blastocyst (tBL), and expanded blastocyst (tEBL) were then determined. Cell cycle intervals t3-t2 (cc2), t4-t3 (s2), t5-t3 (cc3) were calculated. Optimal ranges for kinetics were set based on prior publications [21–24, 27, 28]. The following values were used for optimal timings: t3-t2 (> 5 and ≤ 11.9 h), t4-t3 (≤ 1.0 h), t5 (45–57 h), t5-t3 (9.7–21 h), tSB (< 96.2 h) and tEB (≤ 116 h). Annotations for precise time of blastocyst (tB) and expanded blastocyst (tEB) formation during viewing of TL videos were standardized amongst embryologists. Strict guidelines were established for video footage annotation. Inter-observer variation was controlled for thru peer-review of grading amongst embryologists and discussion of grading criteria at monthly lab meetings.

### Data collection and statistical analysis

Morphokinetic data were exported from the EmbryoScope server. Developmental kinetics of zygotes from epididymal and testicular sperm were contrasted to those from the control group with no obvious female or male factor etiology. The TESE cycles were further subdivided according to the type of azoospermia, TESE-NOA (non-obstructive azoospermia), and TESE-OA (obstructive azoospermia). Continuous data are expressed as means with standard deviations and categorical data as percentages. Univariate analysis using Student’s *t* test, chi square test, and Fischer’s exact test was performed as appropriate to identify parameters influencing blastocyst development as well as clinical pregnancy and implantation rate per embryo transferred. Logistic regression analysis was used to control for potential confounding factors. Analyses were performed after adjusting for age. Odds ratios and 95% confidence intervals were calculated. Statistical analysis was performed using the software package JMP (SAS; Cary, NC, USA). *P* values of < 0.05 were considered to be statistically significant.

## Results

A total of 93 consecutive ICSI cycles performed for 83 couples were included in this study. Observations were made on 594 fertilized oocytes cultured in the EmbryoScope time-lapse chamber. Surgically retrieved sperm from the testis (TESE; *n* = 37) and sperm from percutaneous aspiration of the epididymis (PESA, *n* = 29) were used in 66 cycles. Twenty-seven cycles were performed using fresh ejaculated sperm from men with normal semen parameters where the primary diagnosis for the female partner was tubal factor. This was our “control” sperm group (CS). The average sperm count in the CS group was 81.8 ± 46.2 million/ml with 61.7 ± 17.0% motility. Demographic characteristics for patients having ICSI with CS, PESA, or TESE sperm are shown in Table 1. Female age was not significantly different between PESA (35.6 ± 5.0), TESE (34.6 ± 4.3), and CS (34.1 ± 3.9) groups. AMH levels indicating ovarian reserve were 2.3 ± 1.7, 2.6 ± 2.2, and 2.5 ± 2.1 for patients having CS, PESA, and TESE cycles, respectively. The mean FSH dose and peak estradiol levels also did not differ between women in the groups. Cycle number for women in the CS group was lower (1.07 ± 0.27) when compared to PESA (1.45 ± 0.78, *P* = 0.01) and TESE (1.49 ± 0.60, *P* = 0.001). The mean FSH level of men in the TESE group was 16.4 ± 15.3 mIU, likely indicative of testicular dysfunction and impaired spermatogenesis. Non-obstructive azoospermia (NOA) accounted for 70% of TESE cases (*n* = 26). The age of male partners was younger in the TESE group, 39.1 ± 8.2 as compared to 43.7 ± 9.3 in the PESA cases (*P* = 0.04). Male age in the CS group was 36.6 ± 7.1 and significantly different from the PESA (*P* = 0.001) but not the TESE group.

**Table 1:** Demographics of patients in the CS, PESA or TESE treatment groups

	CS (*n* = 27)	PESA (*n* = 29)	TESE (*n* = 37)	*P* value
Female age	34.1 ± 3.9	35.6 ± 5.0	34.6 ± 4.3	NS
AMH (ng/ml)	2.3 ± 1.7	2.6 ± 2.2	2.5 ± 2.1	NS
FSH dose (mIU/ml)	2513 ± 1290	2550 ± 1685	2041 ± 940	NS
Peak estradiol (pg/ml)	2282 ± 1016	1927 ± 1196	2194 ± 1001	NS
No. cycles	1.07 ± 0.27	1.45 ± 0.78	1.49 ± 0.60	0.001^b^ 0.01^c^
Male age	36.6 ± 7.1	43.7 ± 9.3	39.1 ± 8.2	0.04^a^ 0.001^c^
Azoospermia diagnosis	na	16 s/p vasectomy	6 s/p vasectomy	
		1 ejaculatory duct obstruction	2 epididymal obstruction	
		7 CBAVD	1 Klinefelter	
		5 idiopathic	1 trauma	
			27 idiopathic	
Hormone profile				
LH (mIU/ml)	na	na	11.1 ± 9.2	
FSH (mIU/ml)			16.4 ± 15.3	
Testosterone (ng/dl)			498.3 ± 284.3	

^a^TESE vs PESA ^b^TESE vs CS ^c^PESA vs C TESE for non-obstructive azoospermia (n = 26). *P* value < 0.05 considered to be statistically significant

Table 2 presents clinical outcome data for the three patient subgroups. The clinical pregnancy rate was not different between the three groups (TESE 51.4%, PESA 57.7%, and CS 59.3%). However, the mean number of embryos transferred was slightly higher in the TESE group, 2.27 ± 0.69 (*P* < 0.05) as compared to 1.96 ± 0.60 and 2.0 ± 0.51 in PESA and CS cases, respectively. The implantation rate was lowest with TESE embryos (30.9%) versus PESA (35.3%) and CS (45.1%), but this difference did not reach statistical significance. Only a third of patients in the TESE group qualified for blastocyst stage transfer as compared to 42% and 59% of patients in PESA and CS groups. Subgroup analysis of IR in day 5 transfers also showed no signifcant difference based on sperm type used for ICSI (TESE 55.6%, PESA 65.0%, and CS 72.4%). Lastly, we looked at live birth rates (LBR). A non-significant decrease was observed in LBR with TESE embryos (43.2%) as compared to PESA (57.9%, *P* = 0.26) and CS, respectively (55.6%, *P* = 0.33). Miscarriage rate was also highest with TESE (10.8%) as compared to CS (3.7%, *P* = 0.47). In this series, no significant differences were detected in TESE cases based on type of azoospermia (NOA vs OA). In TESE-NOA cycles, CPR and IR were 46.2% (12/26) and 30.4% (17/56) as compared to 63% (7/11) and 39% (9/23), respectively, in the TESE-OA cycles.

**Table 2:** Clinical outcome data for TESE, PESA, and CS patients

Patient group	Control (CS)	PESA	TESE	*P* value
Retrievals	27	29	37	–
Transfers	27	26	37	–
Patient age	34.1 ± 3.9	35.6 ± 5.0	34.6 ± 4.3	NS
Oocytes retrieved	14.5 ± 8.2	11.6 ± 7.1	14.1 ± 7.4	NS
Mature oocytes	10.4 ± 6.1	8.9 ± 5.4	10.9 ± 5.5	NS
Fertilization rate	79.2%	70.4%	55.2%	< 0.0005^ab^
Fertilized oocytes	8.2 ± 5.4	6.3 ± 3.9	5.8 ± 3.2	< 0.05^b^
Embryos transferred	2.0 ± 0.5	1.96 ± 0.6	2.27 ± 0.69	< 0.05^ab^
Clinical pregnancy rate	59.3% (16/27)	57.7% (15/26)	51.4% (19/37)	NS
Day 3 transfers	18.2% (2/11)	26.7% (4/15)	37.5% (9/24)	–
Day 5 transfers	87.5% (14/16)	100% (11/11)	77% (10/13)	–
Implantation rate	45.1% (23/51)	35.3% (18/51)	30.9% (26/84)	NS
Day 5 only	72.4% (21/29)	65.0% (13/20)	55.6% (15/27)	NS
Live birth rate	55.6% (15/27)	57.9% (15/26)	43.2% (16/37)	NS
Miscarriage rate	3.7%	0%	10.8%	NS
Singleton/multiples	10/5	11/4	12/4*	–

^a^TESE vs PESA ^b^TESE vs CS *P* < 0.05 considered to be statistically significant; *One triplet pregnancy

The mean morphokinetic timings of developing zygotes are shown in Table 3. Embryos derived from testicular sperm appeared to be impaired early on in development. TESE embryos were slower to reach 2, 4, and 8 cells (*p* < 0.05) as compared to PESA embryos. In TESE embryos, time to complete second syngamy (s2) was significantly longer, 4.8 h (95% CI 3.6–6.3) as compared to 2.6 (95% CI 1.8–3.5) and 2.1 h (95% CI 1.6–2.5) for PESA and CS embryos, respectively. The third cleavage event (cc3) was also similarly delayed, occurring approximately 2 h later in embryos originating from testicular sperm, 14.5 h (95% CI 13.4–15.7; *P* < 0.05) versus 12.2 (95% CI 11.0–13.4) and 12.7 h. (95% CI 11.6–13.8) for PESA and CS embryos, respectively. Interestingly, the mean timings for late developmental end points such as compaction, morulation, start of blastulation and progression to expanded blastocyst were not different.

**Table 3:** Comparison of kinetic timings for embryo development in patients using surgically retrieved sperm, PESA or TESE versus control sperm (CS) isolated from semen of non-male factor patients

Patient group	Control (CS) (*n* = 209)	95% CI	PESA (*n* = 175)	95% CI	TESE (*n* = 210)	95% CI	*P* value
Parameter	Mean (h)		Mean (h)		Mean (h)		
t2	26.3	25.7–26.9	26.1	25.4–26.7	27.1	26.5–27.7	< 0.05^a^
t3	36.0	35.0–36.9	35.3	34.2–36.3	36.5	35.6–37.5	NS
t4	38.0	37.2–38.9	37.3	36.3–38.2	39.1	38.2–39.9	< 0.05^a^
t5	48.6	47.2–49.9	46.8	45.4–48.3	48.6	47.2–50.0	NS
t8	58.9	57.4–60.4	58.0	56.3–59.8	60.5	58.7–62.4	< 0.05^a^
CP	67.6	65.3–69.8	63.8	61.3–66.3	69.1	66.5–71.8	< 0.05^a**c**^
tM	91.9	90.3–93.6	92.2	90.3–94.1	91.1	88.8–93.3	NS
tSB	101.2	99.6–102.8	101.4	99.5–103.3	103.5	101.6–105.4	NS
tB	106.2	104.6–107.9	107.3	105.4–109.3	108.5	106.2–110.7	NS
tEB	114.3	112.4–116.2	114.4	112.2–116.6	115.8	113.2–118.5	NS
t3-t2 (cc2)	9.7	8.8–10.7	10.6	9.5–11.7	10.8	9.8–11.8	NS
t4-t3 (s2)	2.1	1.6–2.5	2.6	1.8–3.5	4.8	3.6–6.0	< 0.05^ab^
t5-t3 (cc3)	12.7	11.6–13.8	12.2	11.0–13.4	14.5	13.4–15.7	< 0.05^ab^

*P* value < 0.05 considered to be statistically significant

^a^TESE vs PESA ^b^TESE vs CS ^c^PESA vs CS

In Table 4, we contrast zygotes derived from all three sperm types and their developmental progression to blastocyst. TESE cycles were divided into TESE-NOA and TESE-OA to see if the type azoospermia in the male partner affected embryo growth. A total of 488 zygotes derived from TESE-NOA (*n* = 108), TESE-OA (*n* = 49), PESA (*n* = 145), and CS (*n* = 186) sperm were cultured until day 5/6. Embryonic progression in TESE-NOA embryos was significantly impaired at all stages, from ability to undergo compaction thru morulation, blastocyst formation, and expansion when contrasted to zygotes from CS sperm. Failure to compact was significantly higher amongst TESE-NOA embryos (35.2%; *P* < 0.001) as compared to 4.1% of TESE = OA, 9.0% of PESA and 3.8% of CS embryos. Even when the initial hurdle of compaction was overcome, TESE-NOA embryos still appeared to be compromised. Only 42.9% of compacted TESE embryos developed to expanded blastocyst. This expansion rate was significantly lower than that observed in the comparable CS (65.9%; *P* < 0.001) as well as TESE-OA and PESA subgroups (57.4 and 62.9%, respectively).

**Table 4:** Developmental capacity of CS, PESA, and TESE embryos cultured to blastocyst was compared

Patient group	Control (CS)	PESA	TESE-OA	TESE-NOA	*P* value
Embryos cultured to blastocyst	186	145	49	108	
Compaction failure (%)	3.8 (7/186)	9.0 (13/145)	4.1 (2/49)	35.2^abc^ (38/108)	< 0.001^abc^
Morula/CP (%)	96.2 (179/186)	91.0 (132/145)	95.9 (47/49)	64.8^abc^ (70/108)	< 0.001^abc^
Blastocyst (%)	77.1 (138/179)	73.5 (97/132)	70.2 (33/47)	60.0^ab^ (42/70)	< 0.05^a^, 0.049^b^
Expansion (%)	65.9 (118/179)	62.9 (83/132)	57.4 (27/47)	42.9^abc^ (30/70)	< 0.001^a^, < 0.05^bc^

TESE embryos were further subdivided according to etiology of the azoospermia: OA (obstructive) and NOA (non-obstructive). *P* value < 0.05 considered to be statistically significant ^a^TESE-NOA vs CS ^ b^TESE-NOA vs PESA ^c^TESE-NOA vs TESE-OA

**Fig. 1:**
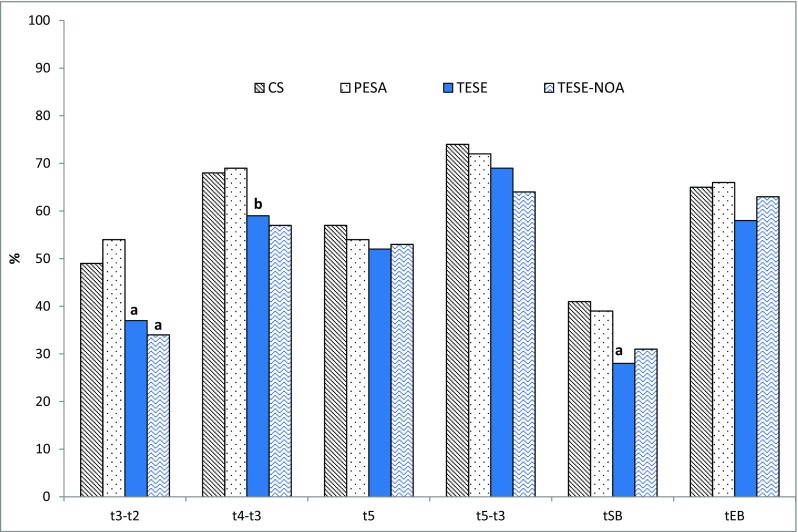
Sperm origin and kinetics of embryo development. Graph shows the percentage of embryos with kinetic timings falling into optimal ranges. Optimal time ranges were defined as follows: t3-t2 (> 5 and ≤ 11.9 h), t4-t3 (≤ 1 h), t5 (45–57 h), t5-t3 (9.7–21 h) tSB (< 96.2 h), and tEB (≤ 116 h). *P* < 0.05 considered to be statistically significant. (a) Significantly different from PESA and CS. (b) Significantly different from PESA

To further characterize embryos derived from different sperm types, we looked at the proportion of embryos that displayed optimal timings for specific kinetic markers based on published selection algorithms as described in the methods section. The optimal ranges for cc2, s2, cc3, t5, tSB, and tEB are shown in Fig. 1. This graph depicts the proportion of embryos falling within the defined range for each kinetic marker and across the different sperm groupings. In a significantly higher proportion of TESE embryos, the timing for transition from 2 to 3 cell (cc2) was out of the optimal time range. The impact of early developmental delay in TESE embryos is further emphasized by the significantly lower proportion of TESE embryos that started blastulation at < 96.2 h. Only 28% of TESE embryos had tSB values that fell within the optimal range as compared to 39% of PESA and 41% CS embryos, respectively (*P* < 0.05).

The relationship between early kinetic variables and the ability to form an expanded blastocyst was also explored. We observed that the odds of an embryo developing into an expanded blastocyst were significantly higher for embryos displaying early kinetics falling in to the defined optimal ranges (Table 5). The three kinetic parameters cc2, cc3, and s2 were independently associated with the embryo’s ability to form an expanded blastocyst in all three sperm treatment groups. The probability of expanded blastocyst formation by TESE-derived embryos showed a five-fold increase when cc2 was in range (OR 5.6, 95% CI 2.7–11.7; *P* < 0.0001). Odds of EB formation by TESE embryos were also significantly increased when cc3 (OR 3.1, 95% CI 1.4–6.8; *p* = 0.005) or s2 (OR 3.3, 95% CI 1.6–6.9; *P* = 0.001) values were in the defined ranges. For PESA-derived embryos, odds ratios of 3.6, 2.2, and 2.9 for EB formation were obtained for in range values of cc2, cc3, and s2, respectively (*P* < 0.05).

**Table 5:** Early kinetic timings and the odds of expanded blastocyst formation for embryos derived from the three different sperm types

Kinetic parameter	Sperm type	Odds ratio EB formation	95% CI	*P* value
cc2 (> 5 ≤ 11.9 h)	CS	2.3	1.2–4.6	0.02
	PESA	3.6	1.8–7.3	< 0.001
	TESE	5.6	2.7–11.7	< 0.0001
cc3 (9.7–21.0 h)	CS	3.3	1.7–6.5	0.001
	PESA	2.2	1.04–4.7	0.04
	TESE	3.1	1.4–6.8	0.005
s2 (≤ 1.0 h)	CS	2.4	1.3–4.6	0.006
	PESA	2.9	1.4–6.1	0.004
	TESE	3.3	1.6–6.9	0.001

*P* value < 0.05 considered to be statistically significant

No relationship was observed between sperm origin and multinucleation. The multinucleation rate in TESE, PESA, and CS embryo groups was 34, 36, and 41%, respectively. Reverse cleavage rate was also similar between the three groups ranging between 10 and 14%. The incidence of direct uneven cleavage (DUC) was however significantly higher after ICSI with sperm retrieved from azoospermic males (*P* < 0.05) compared to normal ejaculated sperm. DUCs were observed in 10% (22/210) and 9% (17/175) respectively of TESE and PESA-derived embryos as compared to only 4% of CS embryos (9/209).

## Discussion

The ability to use surgically retrieved sperm for intracytoplasmic sperm injection has radically altered the prognosis for couples with male factor infertility as the primary diagnosis. The impact of sperm origin on embryonic development and pregnancy outcomes is of great interest in these patients. There are numerous conflicting reports as to the effect of male factor infertility and sperm origin on embryo growth potential, once the fertilization hurdle has been overcome with ICSI. This is one of the first studies to use morphokinetic criteria to objectively characterize paternal influence on embryonic development. TLM allowed us to more precisely identify critical cell cycle endpoints at which TESE-derived embryos exhibited kinetic behaviors distinctly different from PESA embryos as well as embryos of non-male factor origin.

One of the earliest morphologic indicators of embryonic genome activation (EGA) and expression of paternal genes is initiation of E-cadherin mediated cell-cell adhesion, at around the 8-cell stage, leading to compaction [12, 29, 30]. Compaction is a critical transition point for continued embryo development. Disruption in spermatogenesis typically observed with testicular NOA cases may potentially also alter paternal gene expression. In the current work, the compromised developmental capacity of TESE-NOA sperm-derived embryos was most evident from the blastocyst culture data. One in three embryos was unable to undergo embryonic genome activation and initiate compaction. Even after EGA, TESE-NOA embryos continued to show impaired developmental capacity with a significantly lower percentage of embryos progressing to expanded blastocyst. This was not observed with embryos derived from either PESA or TESE-OA sperm.

We are aware of only one other published study using TLM to analyze effect of azoospermia and sperm origin on embryo development. Lammers and colleagues analyzed 48 cycles with surgically retrieved sperm (32 testicular and 14 epididymal cycles) and compared morphokinetic and clinical outcomes to that observed in 556 cycles with ejaculated sperm [31]. In their study, mean timings for cc2, tSC (start compaction), tSB, and tB were significantly delayed with surgically retrieved sperm. Nevertheless, there was sufficient overlap in distribution of timings that individual markers were not found to be predictive of outcome. The authors concluded that morphokinetic analysis did not pinpoint any clinically relevant differences.

We took a slightly different approach in our analysis. The morphokinetic characteristics associated with “good quality” embryos most likely to develop into blastocysts and result in pregnancy have been widely published. A unique aspect of the present analysis was to examine how well the cleavage pattern of embryos from azoospermic males fell into specific “optimal” ranges when compared to counterparts from normospermic males. This offered an objective and perhaps more meaningful way to identify clinically relevant differences in embryo cohorts from different sperm types rather than simply comparing mean kinetic timings. We selected relevant early and late kinetic variables shown to have an association with blastocyst development, implantation potential, or chromosome content based on published literature [21–26, 32]. The two points at which TESE-derived embryos behaved most differently from PESA and CS embryos were at cc2 and tSB. Only 37% of TESE embryos had completed the second cell cycle (cc2) within the optimal time interval as compared to 50% of embryos from the other two groups.

The kinetic marker tSB has been associated with not only blastocyst quality and implantation potential but with chromosome status [22, 27, 32–34]. Campbell and colleagues have proposed tSB as a criterion for predicting risk of aneuploidy. Differences in tSB timing amongst embryos derived from the three sperm groups may therefore be reflective of embryonic competence. In this data set, a significantly higher percentage of PESA (39%) and CS (41%) embryos had tSB timings of < 96.2 h, placing them in the low risk for aneuploidy classification, in contrast to only 28% of TESE embryos. Our own PGS data indicate that slower growing embryos with delayed blastulation have a lower euploidy rate [32].

Non-obstructive azoospermia accounted for 70% of the TESE cases in the current data set. Although the differences were not significant, there appeared to be a distinct trend towards poorer clinical outcomes in terms of CPR, IR, and LBR with testicular sperm. Clinical outcomes after ICSI with testicular versus epididymal sperm have been varied [20]. No doubt the etiology of azoospermia has contributed to the contradictory published reports. Non-obstructive azoospermia is often associated with severe impairment of spermatogenesis, sometimes with only limited regions of active sperm production within the testis. Injection of immature round spermatids in the absence of mature elongated sperm, results in severely impaired embryos with limited growth potential [35, 36]. Immaturity of testicular sperm may express as deficits in sperm centrosome function and sperm aster formation [37]. Sperm methylation necessary for full function as well as the acquisition of motility occurs during sperm transit thru the epididymis [38]. Van Wely et al. showed lower odds of live birth with testicular sperm even in cases of OA with normal spermatogenesis, suggesting that passage thru the epididymis was essential for proper sperm maturation [19]. Testicular sperm also appear to be more prone to aneuploidy [14]. Abnormal sperm chromatin packaging, aneuploidy as well as sperm DNA damage may also negatively influence not only fertilization and early cleavage but also embryonic competence by interfering with embryonic genome activation [12–14, 39–42].

One of the major limitations of this study was its retrospective nature. The etiology of the azoospermic males included in this report could not be adequately controlled for. In addition, we had to identify a “control” group of patients, using normospermic sperm for ICSI, to which we could make meaningful comparisons. These were not age matched controls but rather patients with a primary diagnosis of tubal factor undergoing ICSI during the study interval with no other underlying male or female etiology. These patients also used fresh ejaculated sperm for ICSI, whereas both TESE and PESA cases involved ICSI using frozen sperm. Finally, the number of cases available for analysis was restrictive and did not allow detailed subgroup analysis of NOA vs. OA cycles. Another point of interest was comparison of clinical pregnancy and live birth outcomes with different sperm types based on whether the transferred embryo(s) were in or out of optimal kinetic ranges. Unfortunately, we did not have sufficient known implantation data (KID) to make such an analysis.

In conclusion, embryo assessment with use of TLM, especially for cases with underlying male factor, may be a useful tool in understanding precisely where and how specific sperm defects influence embryonic development. Morphokinetics allow a more objective measure of embryo quality, as well as deselection for negative embryo attributes such as direct uneven cleavage, reverse cleavage, and multinucleation. This study demonstrated a paternal effect on embryo morphokinetics, though the clinical relevance of our findings clearly needs further investigation in a prospective trial with a larger patient base.
